# Evidence for benefit of statins to modify cognitive decline and risk in Alzheimer’s disease

**DOI:** 10.1186/s13195-017-0237-y

**Published:** 2017-02-17

**Authors:** Nophar Geifman, Roberta Diaz Brinton, Richard E. Kennedy, Lon S. Schneider, Atul J. Butte

**Affiliations:** 1 The Manchester Molecular Pathology Innovation Centre, University of Manchester, 3rd Floor Citylabs, Nelson St, Manchester, M13 9NQ UK; 2 Health eResearch Centre, Division of Informatics, Imaging & Data Sciences, University of Manchester, Manchester, UK; 3 Center for Innovation in Brain Science, School of Medicine, Departments of Pharmacology and Neurology, University of Arizona, Tucson, AZ USA; 4 School of Medicine, University of Alabama at Birmingham, Birmingham, AL USA; 5 Keck School of Medicine, University of Southern California, Los Angeles, CA USA; 6 Leonard Davis School of Gerontology, University of Southern California, Los Angeles, CA USA; 7 Institute for Computational Health Sciences, University of California San Francisco, San Francisco, CA USA

**Keywords:** Statins, Alzheimer’s disease, Apolipoprotein E, Cognitive function, Meta-analysis, Clinical trials

## Abstract

**Background:**

Despite substantial research and development investment in Alzheimer’s disease (AD), effective therapeutics remain elusive. Significant emerging evidence has linked cholesterol, β-amyloid and AD, and several studies have shown a reduced risk for AD and dementia in populations treated with statins. However, while some clinical trials evaluating statins in general AD populations have been conducted, these resulted in no significant therapeutic benefit. By focusing on subgroups of the AD population, it may be possible to detect endotypes responsive to statin therapy.

**Methods:**

Here we investigate the possible protective and therapeutic effect of statins in AD through the analysis of datasets of integrated clinical trials, and prospective observational studies.

**Results:**

Re-analysis of AD patient-level data from failed clinical trials suggested by trend that use of simvastatin may slow the progression of cognitive decline, and to a greater extent in ApoE4 homozygotes. Evaluation of continual long-term use of various statins, in participants from multiple studies at baseline, revealed better cognitive performance in statin users. These findings were supported in an additional, observational cohort where the incidence of AD was significantly lower in statin users, and ApoE4/ApoE4-genotyped AD patients treated with statins showed better cognitive function over the course of 10-year follow-up.

**Conclusions:**

These results indicate that the use of statins may benefit all AD patients with potentially greater therapeutic efficacy in those homozygous for ApoE4.

## Background

Alzheimer’s disease (AD) has reached epidemic proportions both in the United States and globally [1–4]. Therapeutics to prevent, delay and treat AD are urgently needed as the epidemic continues to grow with the global aging population. Despite substantial research and development investment in AD, effective therapeutics remain elusive. As of 2008, at least 172 drug development failures in AD therapeutics were identified [5, 6]. In 2012 the Pharmaceutical Research and Manufacturers of America (PhRMA), an industry trade group, acknowledged 101 late-stage clinical trial failures between 1990 and 2012 [7]. There have been no successes since 2002. Drug discovery in neuroscience in general is complicated and uncertain, with overall failure rates greater than 95% for CNS diseases, and very long development programs of 10–15 years from discovery to marketing approval [7].

In the United States, the National Alzheimer’s Project Act (NAPA; US Public Law 111-375) was enacted in 2011 followed by a National Plan to prevent and effectively treat AD by 2025 [1]. To achieve this goal within a decade will require identifying effective therapeutics currently in clinical use and repositioning existing drugs based on conserved pathways and targets of complex diseases [6]. Given the complexity and progressive nature of the disease, it will be necessary to identify phenotypes and genotypes responsive to therapeutic candidates to realize a precision medicine approach to prevention and treatment [8, 9].

Several studies have reported reduced risk for incidence and progression of AD and dementia in statin-treated populations [10–13]. Further, a substantial body of cellular and molecular mechanistic evidence links cholesterol, β-amyloid (Aβ) generation and AD [14–22] and has helped support clinical trials of statins in persons with AD. These trials, however, resulted in no significant therapeutic benefit [23–26]. For example, in one randomized, controlled trial, a 72-week course of treatment with atorvastatin in 640 mild to moderate AD patients did not improve cognitive measures [26]. A second, 18-month, randomized, placebo-controlled trial of simvastatin in 406 participants with mild to moderate AD did not show advantageous clinical effects [24]. In another placebo-controlled simvastatin trial, simvastatin did not significantly alter cerebrospinal fluid levels of Aβ although there was evidence for efficacy in Aβ_1–40_ reduction in persons with “mild” AD [25]. Evidence of dyslipidemia was an exclusion criterion in these trials. A critical, and perhaps determinant, difference between the statin clinical treatment trials and observational studies is that persons in clinical trials were not recruited based on dysregulated cholesterol/lipid homeostasis and in some instances were excluded from enrollment [23, 24]. In contrast, persons receiving statins in observational studies likely had evidence of cholesterol dyslipidemia and would thus be predicted to benefit from therapeutics targeting restoration of cholesterol homeostasis [27].

A key regulator of cholesterol and lipid homeostasis is the cholesterol transporter, ApoE. The ApoE4 allele of the apolipoprotein E gene is associated with higher cholesterol levels [28] and an increased risk of developing AD [29–31]. An average 58–67% of persons participating in clinical trials for AD are ApoE4 positive [32]. Based on these clinical findings and mechanistic analyses indicating that cholesterol and ApoE4 play a role in Aβ burden, metabolism and inflammation in brain, we hypothesize that if statins do have a preventative or therapeutic effect, it would be more evident in persons carrying the ApoE4 allele and that statin use would delay symptoms and progression of AD.

Herein, we investigate whether responders were detectable in multiple patient cohorts of integrated clinical trial data and studies in persons diagnosed with AD as well as prospective observational studies. Meta-analysis of patient-level data integrated from multiple sources can assist in gaining a better understanding of the disease under investigation by enabling comparison of treatments, outcomes and other disease-related patterns. By combining data from many studies, a sufficiently large cohort can be generated and can allow for identification of subgroups who respond better to treatment.

## Methods

### The Alzheimer’s disease integrated clinical studies dataset

Data were drawn from an integrated dataset of Alzheimer’s clinical trials and observational studies described previously [33, 34]. In brief, the datasets consisted of 18 studies from the Alzheimer’s Disease Cooperative Study (ADCS, http://adcs.org) and the Alzheimer’s Disease Neuroimaging Initiative (ADNI, http://www.adni-info.org) conducted from 1993 to 2012 to analyze the decline on the Alzheimer’s Disease Assessment Scale—cognitive subscale [35] (ADAS-cog), the Clinical Dementia Rating—Sum of Boxes [36] (CDR-SB) scale and the Mini-Mental State Examination [37] (MMSE) over time. The integrated dataset includes demographics information, cognitive assessments, ApoE genotyping, concomitant medications information and blood test data for a total of 4574 participants, and 25,164 encounters. All diagnoses of AD were based on National Institute of Neurological and Communicative Disorders and Stroke/Alzheimer’s Disease and Related Disorders Association criteria [38]. Statin use was captured from the concomitant medication logs using the following search terms: “simvastatin”, “fluvastatin”, “atorvastatin”, “rosuvastatin”, “lovastatin”, “pravastatin”, “pitavastatin”, “Crestor”, “Lipitor”, “Lescol”, “Mevacor”, “Pravachol”, “Zocor” and “Livalo”.

#### Re-analysis of a simvastatin trial

**Fig. 1:**
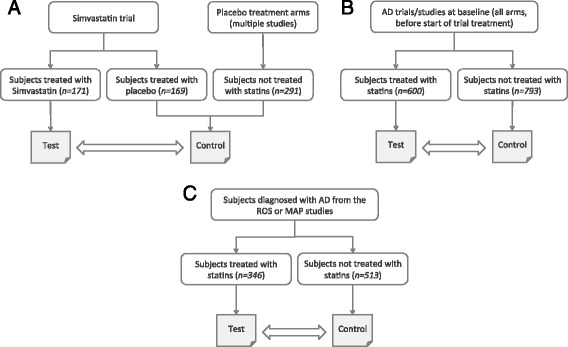
Analyses workflows. **a** Re-analysis of simvastatin trial. **b** Analysis of continual use of statins from multiple AD trials and studies. **c** Analysis of statin use from the ROS/MAP studies. *AD* Alzheimer’s disease, *MAP* Memory Aging Project, *ROS* Religious Order Study

A simvastatin-treated group [24] consisting of 171 subjects with a determined ApoE genotype who were treated with simvastatin 20 mg/day for 6 weeks and then 40 mg/day for the remainder of 18 months (Fig. 1a) formed our treatment (test) group. To increase our ability to detect treatment effect, subjects treated with placebo who met our selection criteria were pooled to create a larger comparator arm. A total of 460 AD subjects assigned to placebo-treated groups from six trials included in the dataset, with no known concomitant statin use, with a determined ApoE genotype, and with at least one assessment on the ADAS-cog (at baseline or thereafter) were used for the comparator arm. These trials included ADCS studies evaluating the effects of simvastatin [24], docosahexaenoic acid supplementation [39], estrogen replacement therapy [40], B vitamin supplementation [41], rofecoxib or naproxen [42] and prednisone [43], selected for their inclusion of a placebo-treated arm, availability of ApoE genotype data, availability of evaluations at matching time points and for having similar baseline mean ADAS-cog scores.

#### Analysis of continual statin use

The effect of continual statin use (prior to study recruitment) on ADAS-cog scores was examined (at study baseline, before commencement of the trial treatment) in AD subjects (*n* = 1393) pooled from ADNI [44] and ADCS trials evaluating the effects of docosahexaenoic acid supplementation [39], B vitamin supplementation [41], huperzine A [45] and valproate [46], selected for their availability of a baseline measurement of ADAS-cog scores and for including both statin users and nonusers. Of the 1393 subjects, 793 subjects had no known concomitant statins use and were used as the control group and a total of 600 subjects had known use of statins at baseline (Fig. 1b and Table 1B); 273 subjects were treated with atorvastatin, 178 subjects were treated with simvastatin, 28 subjects were treated with rosuvastatin, eight subjects were treated with fluvastatin, 68 subjects were treated with lovastatin and 45 subjects were treated with pravastatin. Drug doses varied between subjects and information regarding duration of treatment was not available.

**Table 1:** Baseline demographic information for each of the treatment groups in the three analyses

	A. Simvastatin trial re-analyses			B. Continual use of statins (trials from the integrated dataset)		C. Continual use of statins (ROS/MAP dataset)	
	Simvastatin arm	Original placebo arm	New (pooled) placebo arm	Statin users	Statin nonusers	Statin users	Statin nonusers
Number of subjects	171	169	460	600	793	346	513
Age (mean, years)^a^	73.3 ± 9.8 SD	74.5 ± 8.9 SD	75.0 ± 8.6 SD	76 ± 7.5 SD	76.7 ± 8.4 SD	81.8 ± 6.6 SD	82.5 ± 6.7 SD
Gender (% female)	57.7	60.5	58.9	49.8	59.5	74.3	69.0
Education (mean, years)^a^	14.5 ± 2.9	14.2 ± 3.3 SD	14.2 ± 3.3 SD	14.0 ± 3.1 SD	14.0 ± 3.1 SD	15.9 ± 3.6 SD	16.3 ± 3.8 SD
Race (%)^a^	91.7 white and 5.8 black or African American	93.2 white and 5.6 black or African American	91.2 white and 5.4 black or African American	91.8 white and 5.7 black or African American	89.9 white and 6.9 black or African American	91.0 white and 7.5 black or African American	94.9 white and 4.9 black or African American
ApoE4 carriers (%)^a^	61.8	55.0	60.2	66.7	62.1	37.5	31.8
Mean baseline cognitive measure	24.2 ± 9.4 SD (ADAS-cog)	24.0 ± 10.0 SD (ADAS-cog)	23.9 ± 9.3 SD (ADAS-cog)	23.5 ± 9.6 SD (ADAS-cog)	24.9 ± 9.4 SD (ADAS-cog)	25.3 ± 5.3 SD (MMSE) –0.5 ± 0.8 SD (global cognitive scores)	25.4 ± 4.0 SD (MMSE) –0.55 ± 0.8 SD (global cognitive scores)

*A* demographic information for the simvastatin trial re-analyses treatment arms, *B* demographic information for treatment groups used in the analysis of continual use of statins (in five studies from the integrated clinical studies dataset), *C* demographic information for treatment groups used in the analysis of statins use in the ROS/MAP datasets

*ADAS-cog* Alzheimer’s Disease Assessment Scale—cognitive subscale, *MAP* Memory Aging Project, *MMSE* Mini-Mental State Examination, *ROS* Religious Order Study, *SD* standard deviation

^a^Percentage of those subjects with relevant information available

### The Religious Order Study/Memory Aging Project dataset

A dataset comprising data combined from Religious Order Study (ROS) and Memory Aging Project (MAP) [47, 48] was obtained from the Rush Memory and Aging Project [49] and accessed through the Sage Bionetworks Synapse portal [50]. In brief, this dataset included 3103 subjects of which 859 were classified as having a probable or highly probable diagnosis of AD at some point throughout the study’s follow-up. AD diagnosis was established through review of self-reported questions, neurological examinations (when available), cognitive testing and interviews of participants. Statin users were defined as those subjects who reported ever using statins [51]; duration of statin use was not available. Use of nonstatin lipid-lowering drugs was also recorded and included the use of any of the following: ezetimibe, fenofibrate, gemfibrozil, niacin, colesevelam HCl, omega-3-acid ethyl esters, cholestyramine, fenofibric acid, colestipol and probucol. All medications taken in the 2 weeks prior to evaluation were reported by participants; containers were visually inspected and coded using the Medi-Span Drug Data Base system. For each subject, baseline was individually defined as the first visit at which statin use was reported. MMSE [37] scores, global cognitive scores, demographics data and information regarding other relevant medical conditions such as heart conditions or stroke were collected and used in this analysis. The global cognitive scores were computed by combining the results of 19 cognitive tests used to assess five domains of cognitive function (episodic, semantic and working memory, perceptual speed and visuo-spatial ability). Raw scores from the individual tests were converted to *Z* scores and averaged to yield a global cognitive summary score, which was used in our analysis [52].

### Statistical analyses

For each of the analyses, baseline characteristics were compared between the treatment and control arms; a chi-squared test was used for categorical variables (gender, ethnicity and ApoE4 carriers) and *t* tests were used for continuous data (age, number of education years and baseline cognitive scores). A level of significance of 5% was used.

In the re-analysis of the ADCS simvastatin study (Fig. 1a), differences in ADAS-cog scores between treatment and control groups were evaluated using a mixed-effects model implemented in R (using the lme4 package [53]) to test for differences in the slopes (rate of change) of the ADAS-cog score between the treatment and placebo groups over the entire follow-up period (five time points: baseline, 3, 6, 12 and 18 months). The model included the group effect, the visit (time) effect and group-by-visit interactions, with ApoE genotype, race, age, education level, the individual studies each subject originated from and gender as covariates. The mixed-effects model was selected because it utilizes data from all participants (rather than just completers), minimizes bias and better controls for type I error in the presence of missing data [54].

For analysis of continual use of statins in a cohort of subjects pooled from multiple Alzheimer’s clinical trials and studies (at baseline), linear regression was used and included ApoE genotype, race, age, education level, the individual’s study and gender as covariates (Fig. 1b).

In the analysis of the ROS/MAP dataset, a Cox proportional hazards model was used to estimate the effect of statin use on incidence of AD, excluding subjects with an AD diagnosis at baseline, and was adjusted for age, gender, race, education level, ApoE genotype and any diagnosis of heart conditions or of stroke. A chi-squared test was used to calculate differences in AD prevalence between statin users and nonusers.

A mixed-effects model was applied in the evaluation of differences in change in MMSE or global cognitive scores between statin users and nonusers over time (Fig. 1c). Here, the model included the group effect, the visit (time) effect and group-by-visit interactions, and was adjusted for age, gender, race, education level, ApoE genotype, diagnosis of heart conditions and diagnosis of stroke.

## Results

We first evaluated a possible therapeutic effect of simvastatin on decline in ADAS-cog scores of persons diagnosed with AD by analysis of an integrated dataset of failed Alzheimer’s clinical trials. For this analysis, subjects treated with simvastatin (from the ADCS simvastatin trial [24], the only statin trial available in the dataset) were compared with subjects with no known statin use (from multiple trials) who were pooled into a single comparator arm (see Methods).

The demographics of the pooled comparator arm were very similar to those in the original comparator arm from the simvastatin trial (Table 1A). The main difference between the original and the pooled comparator arms was the percentage of ApoE4 carriers, 60.2% in the pooled comparator arm and 55.0% in the original comparator arm. Other than age, no differences in baseline characteristics or baseline ADAS-cog were found between the simvastatin and pooled placebo arms (Table 1A). Age was statistically higher in the pooled placebo arm relative to the simvastatin arm (*p* = 0.03), but in the ApoE4/ApoE4 subgroups no differences in age or any other baseline characteristics were found.

**Fig. 2:**
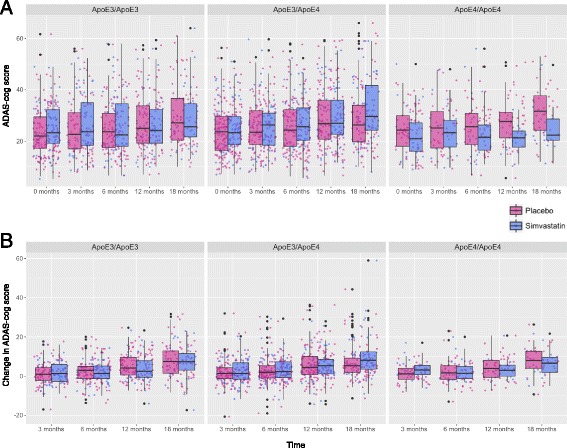
ADAS-cog scores in subjects treated with simvastatin or placebo. **a** ADAS-cog scores over time in different genotype subgroups. **b** Change in ADAS-cog scores from baseline in different genotype subgroups. *ADAS-cog* Alzheimer’s Disease Assessment Scale—cognitive subscale, *ApoE* apolipoprotein E

Comparison of ADAS-cog scores between the two treatment groups over time revealed no significant differences. However, when comparisons were conducted for each subgroup of ApoE genotypes separately (Fig. 2), some differences were observed (although not statistically significant). In subjects with the ApoE4/ApoE4 genotype, those treated with simvastatin showed lower (better) ADAS-cog scores than those treated with placebo; an average difference of 6.1 and 7 points at 12 and 18 months of treatment, respectively. These differences were not statistically significant but indicated a trend.

Next, we compared the effects of chronic, or continual, use of various statins on ADAS-cog scores. Subjects (*n* = 1393) pooled from all treatment arms of five studies (from the integrated clinical trials and studies dataset) with a baseline assessment on the ADAS-cog were grouped by their statin use status (users vs nonusers) prior to study recruitment, and were examined as a prospective observational cohort. Gender was significantly different between statins users and nonusers (with fewer females in the statin users group, *p* < 0.05), while no differences were found in age, race, education or % ApoE4 carriers (Table 1B).

**Fig. 3:**
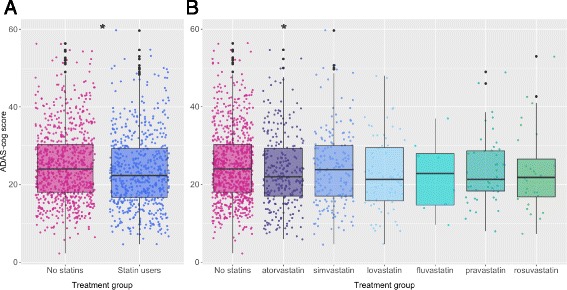
Continual use of statins vs placebo. **a** ADAS-cog scores at baseline. **b** Continual use of different statins vs no statin use. *Significant differences between statin users and nonusers (*p* < 0.05). *ADAS-cog* Alzheimer’s Disease Assessment Scale—cognitive subscale

Comparison of ADAS-cog scores for subjects treated with statins with those with no known use of statins (at baseline of their respective study) revealed that statin users were significantly less cognitively impaired than those not treated with statins (with a mean score of 24.9 ± 9.4 SD in nonusers and a mean score of 23.5 ± 9.6 SD in statin users, *p* < 0.01; Fig. 3a). In the evaluation of individual statins, this effect was significant with the use of atorvastatin (mean scores of 23.5 ± 9.6 SD, *p* = 0.026; Fig. 3b) and marginally nonsignificant with the use of lovastatin (mean scores of 22.4 ± 9.7 SD, *p* = 0.07)—both of these are lipophilic statins predicted to have high blood–brain barrier penetration [55]. No correlation was found between ADAS-cog scores and total cholesterol or triglyceride levels.

To validate our findings in another cohort of subjects, 859 AD subjects were identified in the ROS and MAP research cohort datasets. Of these AD subjects, 513 subjects had no known use of statins while 346 reported using statins (Table 1C). No differences in age, gender, race, education, MMSE or global cognitive scores were found between the statin user and nonuser groups at baseline (Table 1C); the statin nonuser group had fewer ApoE4 carriers. Similarly, there were no baseline differences between the ApoE4/ApoE4 subgroups of statin users and nonusers. No correlations were found between MMSE or global cognitive scores and total cholesterol levels in either statin users or nonusers.

**Fig. 4:**
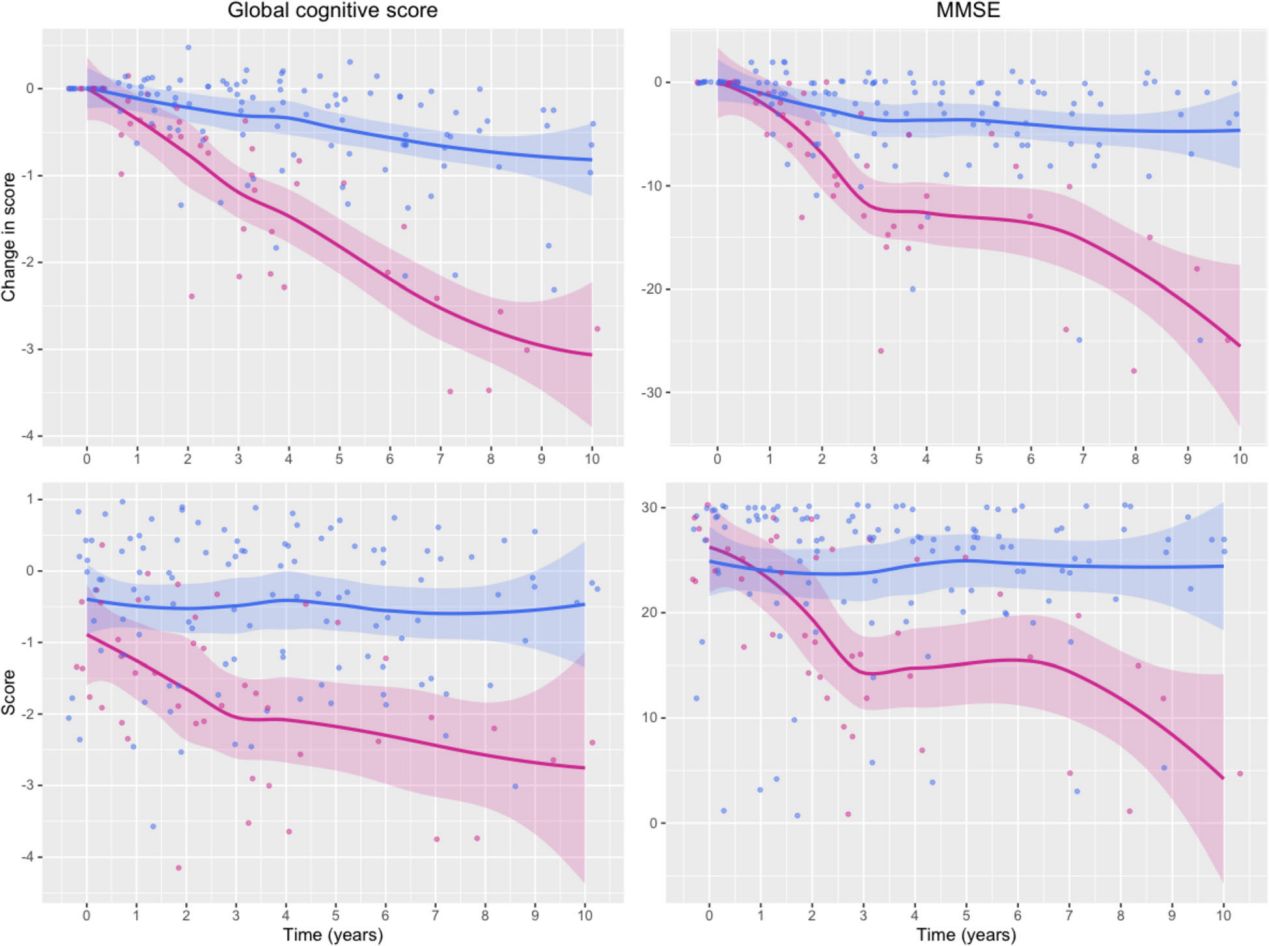
Effect of continual use of statins on global cognitive scores and MMSE scores. *Top panels*: illustration of differences in the change in scores (from baseline) in statin users and nonusers. *Bottom panels*: illustration of differences in scores between statin users and nonusers. *Left-hand panels*: global cognitive score. *Right-hand panels*: MMSE score. Plot lines were smoothed using the locally weighted scatterplot smoothing (LOESS) method; *shaded areas* represent the 0.95 confidence interval (*blue line*, statin users; *red line*, statin nonusers). *MMSE* Mini-Mental State Examination

In a Cox proportional hazards model (of 2570 subjects from the ROS/MAP dataset, after excluding those with a diagnosis of AD at baseline), statin use was associated with lower risk of AD (HR = 0.8; 95% CI 0.68, 0.95; *p* < 0.01). At the end of follow-up, the prevalence of AD in subjects using statins was 24.8% while in subjects with no known use of statins (at start or throughout follow-up) the prevalence of AD was 30.7% (*p* < 0.0005). Comparison between all AD statin users and nonusers revealed no statistically significant differences in cognitive function over time. However, in ApoE4/ApoE4 AD subjects (*n* = 24), those who were treated with statins had significantly better cognitive function over the course of 10-year follow-up, demonstrating significantly slower decline in MMSE and global cognitive scores over time (*p* < 0.01; Fig. 4). The use of nonstatin lipid-lowering drugs had no significant effect on either cognitive measure; however, there were only three subjects in the ApoE4/ApoE4 AD nonstatin lipid-lowering drug user group.

## Discussion

Clinico-pathological studies have demonstrated an extended preclinical phase of the disease, with pathological processes estimated to begin up to 15–20 years prior to the onset of clinical symptoms [56]. Cognitive impairment occurs subsequent to a continuum of multiple indicators of disease progression that precede cognitive dysfunction and conversion to AD [57]. Thus, prevention or treatment at the early or even preclinical stages of the disease are extremely important and most likely have the greatest chances for success [58].

Our re-analysis of data from a randomized, double-blind, placebo-controlled trial examining the effect of simvastatin on progression of symptoms in individuals with mild to moderate AD [24] suggested by trend (but not supported by statistical testing) that statins may have some therapeutic effect. By pooling data from multiple trials, a pattern of lower cognitive impairment—specifically in subjects with an ApoE4/ApoE4 genotype—was demonstrated in the simvastatin-treated arm. Combining subjects from different trials, with different selection criteria, is likely to introduce some level of noise and patient variability. However, combining data from multiple studies has the potential to lead to new discoveries and insights [59–62]. By increasing the overall number of subjects being analyzed (while controlling for similar baseline characteristics and cognitive scores), and by substantially increasing the number of ApoE4/ApoE4 genotyped subjects in the dataset, we were able to demonstrate a trend that was not detectable previously. Because ApoE4 is known to be associated with higher cholesterol levels and has been implicated in AD-related process such as Aβ burden and inflammation, it may act as a biomarker for subjects who would benefit to a greater extent from use of statins. Lack of statistical significance for the change in ADAS-cog scores over time between the two treatment groups could be explained by the small number of subjects with the ApoE4/ApoE4 genotype for which scores were available at baseline as well as at 12 or 18 months; alternatively, it may be due to lack of effect. The results of this analysis hinted at a possible therapeutic effect of statins in AD, and formed the basis for further investigation of larger datasets.

To further investigate a possible beneficial effect of statins, the effect of continual use of statins was evaluated by grouping subjects from multiple studies and clinical trials into effectively a retrospective observational cohort. Results of this analysis revealed that statin users had better cognitive scores than nonusers; and this effect was somewhat more evident with the use of some lipophilic statins (atorvastatin and lovastatin). It has been suggested previously that these specific statins may be more effective in the treatment or prevention of cognitive decline, at least in part, due to their ability to cross the blood–brain barrier [55, 63]. However, evidence from this study is insufficient to support the conclusion that the beneficial effect of statins on ADAS-cog scores is limited to lipophilic statins.

A shortcoming of this dataset arises from the variation of dosages and lack of information regarding duration of statin treatment. Thus it was not possible to differentiate between subjects who may have been using statins for several years and those who had only been using statins for several months, and the length of treatment needed to see an effect on cognitive decline could not be assessed. Furthermore, it was unclear to what extent the concomitant medication logs of the trials used in this study were complete.

Cognitive measures were also evaluated in a second cohort of AD subjects, established from the ROS and MAP observational studies. Significant differences in the change in cognitive scores over the course of 10-year follow-up were found between ApoE4/ApoE4 genotyped subjects with known statin use versus those with no known statin use, further demonstrating that statin treatment may lower cognitive decline. Previous analysis of the ROS study alone found no differences in change in cognitive scores between statin users and nonusers [51]; however, these were not evaluated in subjects homozygous to ApoE4 alone. When analyzing subjects with an ApoE4/ApoE4 genotype, from a larger cohort comprising both the ROS and MAP studies, these differences were detected. Our analysis of incidence rates of AD in all statin users and nonusers from the ROS/MAP dataset revealed significantly lower risk for AD in statin users. These findings are consistent with findings from the GEMS study in which statins were demonstrated to slow the rate of cognitive decline and delay the onset of AD in healthy subjects [64], as well as the Rotterdam study in which use of statins was associated with decreased risk for AD [13]. Based on our initial findings, a parallel independent validation analysis of Medicare records was conducted; results of those analyses are consistent with findings reported herein and extend our findings to indicate reduced incidence of AD in statin users in both sexes and multiple races [65].

The ROS/MAP observational dataset also has shortcomings. In this dataset, 859 AD subjects were identified; while ApoE genotype data were available, the number of subjects with an ApoE4/ApoE4 genotype was relatively small (24 AD subjects had a confirmed ApoE4/ApoE4 genotype, 16 statin users and eight nonusers). Additionally, as with the continual statin use trial dataset, information regarding the length of time for which subjects were treated with statins or the dosage was not available. Future work should focus on establishing which specific statin, dosage and duration of treatment exert the greatest beneficial effect. It is possible that a relatively long duration of treatment (of many years) may be required. This would also explain in part why clinical trials, which do not tend to run for such extended durations, have failed to demonstrate a protective or therapeutic effect of statins in AD.

While no correlations were found between cholesterol levels and ADAS-cog scores, MMSE or global cognitive scores, it is possible that statins may exert a beneficial effect on cognitive decline via a mechanism associated with the restoration of cholesterol homeostasis. This would also account for the difference in the significance of the beneficial effects observed in the analyses of our prospective datasets vs the re-analysis of the clinical trial (where subjects with dyslipidemia were excluded from the simvastatin-treated arm). Evidence has suggested that rs3846662, a polymorphism in the HMG-CoA reductase gene, is a genetic modifier for the risk, age of onset and conversion of AD, and to a greater extent in ApoE4 carriers [66], supporting the involvement of HMG-CoA reductase in the effect of statins in AD. Additionally, lovastatin has been show to lower brain cholesterol in normal but not ApoE-deficient mice [67]. However, it may also be the case that statins can affect cognitive decline by operating on targets other than the HMG-CoA reductase enzyme for which they are targeted. There is a growing body of preclinical evidence supporting targets for statins in the brain including nonamyloid mechanisms as well as targets that are independent from HGM-CoA reductase [68, 69].

While various factors were controlled or adjusted for, it is possible that other confounders or biases could account for the differences found between statin users and nonusers in the analyses presented here. For example, if statins are more likely to be prescribed to patients with good cognition, reverse causation could lead to a misleading beneficial association. Additionally, other differences in prescription practices, indications for statin use and adherence to treatment may also confound our analyses [70].

Because it is difficult to draw definitive conclusions from observational studies, a randomized controlled trial is preferred for the investigation of the association between statin use and dementia and AD. Past trials, however, have either excluded patients with dyslipidemia or were not focused on patients with dementia or AD. For example, two large-scale, randomized, placebo-controlled trials of simvastatin [71] and pravastatin [72, 73] in subjects with or at risk for cardiovascular disease showed no effect on cognitive function. In both of these trials, however, cognition was only a tertiary endpoint and subjects with dementia at baseline were excluded. It may also be the case that earlier exposure to statins is required in order to achieve a positive effect on cognition, while exposure later in life, and closer to the onset of dementia and AD, would be less likely to have a beneficial effect [74]. While a much larger and extensive clinical trial, focusing on ApoE4 carriers, will be required to validate our findings, these, along with those reported previously, provide the foundation to design a precision medicine approach to statin therapy for AD. A trial that incorporated the extensive existing data on efficacy of statins to reduce risk or modify the course of disease, duration of treatment, sex and ethnicities differences, stage of disease progression and pharmacogenomic response to statins would likely enhance probability of success and reduce both participant number and trial duration.

## Conclusions

Overall, our results indicate that the use of statins may benefit all AD subjects and may be most beneficial in subjects with an ApoE4/ApoE4 genotype. This work provides an example for utilizing existing large patient-level datasets, and for use of a precision medicine approach to evaluate the effect statins may have on cognitive impairment and the identification of subpopulations of subjects who will most benefit from such treatment. Going forward, key issues to be explored are genotypes and phenotypes most appropriate for statin therapy as a preventive or disease-modifying therapy, and statins with greatest therapeutic efficacy in ApoE genotypes for preventing or delaying AD.

Results of our analyses contribute to a growing body of evidence indicative of therapeutic benefit of statins within a responder subset and thus have the potential to impact the risk and course of AD.

## Abbreviations

AD: Alzheimer’s disease
ADAS-cog: Alzheimer’s Disease Assessment Scale—cognitive subscale
ADCS: Alzheimer’s Disease Cooperative Study
ADNI: Alzheimer’s Disease Neuroimaging Initiative
ApoE4: Apolipoprotein E, allele 4
CDR-SB: Clinical Dementia Rating—Sum of Boxes
GEMS: Ginkgo Evaluation of Memory Study
HMG-CoA: 3-Hydroxy-3-methyl-glutaryl-coenzyme A
MAP: Memory Aging Project
MMSE: Mini-Mental State Examination
NAPA: National Alzheimer’s Project Act
PhRMA: Pharmaceutical Research and Manufacturers of America
ROS: Religious Order Study
SD: Standard deviation
